# Experimental Investigation of Hybrid Beams Utilizing Ultra-High Performance Concrete (UHPC) as Tension Reinforcement

**DOI:** 10.3390/ma15165619

**Published:** 2022-08-16

**Authors:** Ibrahim Y. Hakeem, Muhammad Kalimur Rahman, Fadi Althoey

**Affiliations:** 1 Department of Civil Engineering, Najran University, Najran 55461, Saudi Arabia; 2 Interdisciplinary Research Center for Construction and Building Materials, King Fahd University of Petroleum & Minerals, Dhahran 31261, Saudi Arabia

**Keywords:** UHPC, flexural strength, hybrid NC-UHPC beams

## Abstract

Ultra-high performance concrete (UHPC) is a new generation concrete with extremely high tensile and compressive strength, high durability, and ductility. UHPC offers tremendous opportunities for use in new thin and slender structural concrete elements and repair of existing concrete structures and has an excellent potential to replace conventional steel reinforcement in normal concrete (NC) members. This paper investigated the potential application of a hybrid NC-UHPC beam using a thin UHPC layer on the tension face to cater to tensile stresses, eliminating the need for passive steel reinforcement. Four-point flexural load tests were performed on 24 composite beams with a thin UHPC layer overlaid with NC. The parameters considered include the thickness of the UHPC layer, depth, and span of the beam. A linear behavior categorizes the flexural behavior of the hybrid NC-UHPC beam up to the ultimate load, after which the hybrid beam shows a non-brittle failure, and softening ensues associated with cracking, increased deflection, and loss of load resisting capacity. The unfinished top surface of the UHPC layer and the overlying NC developed a full composite action without any slip. It was found that a two-day self-curing of the UHPC layer was found to be essential for the development of a strong bond between the layers. The random dispersion and orientation of steel fibers in the UHPC can lead to a decreased tensile response for larger hybrid NC-UHPC beams. The experimental results validate the potential of hybrid NC-UHPC beams as an attractive, structurally feasible, and alternative sound form of construction in terms of their high flexural strength and corrosion-free service life. The proposed unreinforced hybrid system could be used in the construction of precast beams and slabs for residential as well as industrial buildings. Further research, including full-scale load testing of the hybrid beam, is needed prior to practical applications.

## 1. Introduction

The advancement of steel fiber reinforced ultra-high performance concrete (UHPC) with a flexural strength reaching 30 MPa has created tremendous opportunities for exploring hybrid NC-UHPC concrete members. Taking the benefit of the outstanding tensile strength of UHPC, the substitution of the traditional steel reinforcement needed in the tension zone of the concrete beams could be a strong alternative. If feasible, such construction has a substantial potential for implementation in harsh environments; wherever the deterioration associated with corrosion of steel reinforcement is considered the main issue for the durability of normal concrete (NC) structural members. The practice of reinforcing structural members without steel reinforcement in a corrosive environment is extremely desirable. There is a possibility that such a hybrid design could provide an economic advantage. This research presents the outcomes of an investigation executed to evaluate the flexural behavior of two-layer composite NC-UHPC beams without flexural reinforcement. The hybrid NC-UHPC beam is a two-layer composite beam in which the upper part, including the compression zone, is made of normal concrete and a thin layer of UHPC forms the lower part in the tension zone. The UHPC layer replaces the steel reinforcement needed in NC beams and caters to the tensile stress generated at the bottom of the flexural member.

Numerous research papers were dedicated to the evaluation of the durability and strength performance of UHPC. These research findings confidently established the outstanding characteristics of UHPC due to its dense microstructure, including a very high strength > 150 MPa with a linear elastic behavior up to 80–90% of the ultimate load. UHPC offers very a high flexural strength of 48 MPa, high bond strength to the reinforcing bars and fibers, resistance to blast load, impact resistance, enhanced fatigue behavior, reduced pore volume and pore size resulting in very low water absorption, low chloride diffusion, reduced porosity and permeability, and very high durability [1,2,3,4,5,6,7,8,9,10,11,12,13,14,15,16]. Hakeem et al. [17] examined the potential utilization of UHPC under cyclic exposure to aggressive media. The basic durability and mechanical performance of UHPC made from imported pre-packed commercial UHPC material (Ductal) were evaluated, affirming the claim that UHPC possesses exceptional mechanical characteristics in addition to high durability performance in severe environments. However, UHPC has some disadvantages, including its high cement content, relatively higher cost, the need for organized mixing of constituent materials, and non-feasibility of making the top surface of UHPC smooth or leveled after casting using the traditional methods [18,19,20,21,22,23]. The influence of small size steel fibers, in addition to heat cycling, on the fracture characteristics of UHPC was studied [17]. Additionally, the influence of repeated/cyclic loads, amount of steel fiber, and curing methods on the compressive behavior, as well as modulus of elasticity of UHPC, were also investigated [18,19,20]. The potential for UHPC mixes using the industrial waste product and naturally available materials as fractional substitutes for natural fine dune sand and micro silica was investigated, and the mixes developed met the required flow (above 180 mm) and strength (above 150 MPa) properties [21]. Development of UHPC mixes using locally available materials in the eastern region of Saudi Arabia with lower amounts of silica fume and steel fibers to reduce the unit cost was reported [20,21,22,23].

UHPC, with its exceptional durability, high compressive and tensile strengths, and improved ability to form, offers a host of chances to rethink the production, rehabilitation, and maintenance of roads and bridges. UHPC has a great potential to provide solutions for the development of new areas of applications and new construction systems that hitherto were not possible. Several innovative applications of UHPC were reported in the literature. Some innovative applications include using UHPC in the production of train station canopy by Perry and Zakariasen [24] and the use of UHPC in large bridge girders by Graybeal [25] and Theresa et al. [26]. Pedestrian bridges made of UHPC were also built and reported [27,28].

Another important area of UHPC applications is hybrid systems. In recent years, several research papers have been published on the hybrid use of UHPC in structural applications. Significant efforts were made to develop a new hybrid application of UHPC that can dramatically improve the long-term performance of engineered structures such as hybrid steel-UHPC, UHPC-wood, and NC-UHPC structural members.

The key aspect for the successful performance of a hybrid system is the adequacy of the bonding between the layers of the composite system. Bassam et al. [29] investigated the composite action of a UHPFC-NC hybrid system, and it was found that the UHPC overlay developed a high bond strength and a high impermeability at the interface with the NC substrates with the sandblasted substrate surface, exhibiting the best interfacial mechanical bonding. The slant shear test showed strong bonding, with failure at the interface occurring due to cracking and crushing in the NC substrate or failure of the NC substrate without interfacial separation or de-bonding. Azad and Hakeem [30,31] investigated a novel beam design using UHPC and introduced the concept of UHPC bars as tensile reinforcement. Flexural behavior assessment without conventional reinforcement proved adequate structural performance. The hybrid beams reinforced with ready-made bars of UHPC subjected to four-point flexural loading showed that the UHPC bars developed a flexural capacity of around 30 MPa at ultimate load without any slip at the interface between normal concrete and UHPC bars. Hybrid NC-UHPC beams with UHPC bars demonstrate a ductile mode of failure associated with a softening part after peak load, with deflection increasing as the residual strength decreases. The flexural performance of novel hybrid hollow-core slabs with UHPC layers as a new hybrid slabs construction, in which a central part of the hybrid hollow-core slab portion of normal concrete sandwiched between the top and bottom film of UHPC, was also investigated by Azad and Hakeem [32] to test the flexural capability. Yuen et al. [33] presented a bridge deck concept using a composite NC-UHPC structural system. Composite NC-UHPC beams 1.2 m long, 250 mm wide, and 150 mm in depth with UHPC substrate of varying thicknesses (50–70 mm) overlaid by an NC layer were tested under 4-point flexural loading. The beams were tested with and without steel reinforcement in the UHPC layer. The non-reinforced NC-UHPC beams increased the strength and stiffness of the beam significantly. Failure modes including flexural, shear, and interface cracking were observed. Shear cracking resulted in beams with 70 mm thickness. Numerical simulation using ATENA software captured the experimental response. Liu et al. [34] investigated the shear bond performance of the NC-UHPC interface with different types of interfaces and casting sequences. Most of the specimens failed by damage to the NSC layer at the interface. The casting sequence also had a strong influence on the bond strength. Similarly, Guan et al. [35] studied the effects of castellated key dimension and dowel rebar on the shear behavior of the UHPC-NSC interface. They found that the effect of the cohesion between the UHPC and NSC on the interfacial shear resistance was very limited and could be ignored. Furthermore, they reported that when there was a dowel rebar in the joint specimens, the specimens had shear resistance with post-peak residual loads. Rezakhani et al. [36] investigated the effect of fiber size and shape on the responses of UHPC under quasi-static loading conditions. The tensile strength of UHPC is strongly influenced by the fiber shape and size.

In hybrid NC-UHPC beams, the bond between the ordinary concrete and the purposely uneven surface of the UHPC stratum provides acceptable interfacial shear resistance to ensure combined achievement under loading. The flexural strength of almost 30 MPa achieved in UHPC offers a chance to design a hybrid structure with NC-UHPC structural flexural members, where UHPC could substitute passive steel reinforcement to achieve the needed flexural strength. If structurally achievable, such a design can provide meaningful benefits in corrosive environmental situations, where steel corrosion is a major durability concern. The main aim of the current study was to present the findings of a comprehensive experimental and analytical study on the flexural behavior of hybrid concrete beams made of NC with a UHPC sublayer in the tension zone. The investigations conducted on small size specimens showed that the proposed concept has a high potential for structural applications. Further investigations on large-size beam and slab specimens are needed.

## 2. Experimental Program

The experimental program conducted in this research included four-point flexural load tests on 24 simply supported hybrid NC-UHPC beams. Flexural strength, cracking, and failure modes of hybrid beams cast with a UHPC bottom layer without steel reinforcement were investigated with the objective of finding an alternative form of beam or slab construction that could eliminate the need for conventional steel reinforcement, thereby providing high durability in corrosive environments.

### 2.1. Materials and Mix Design

**Figure 1:**
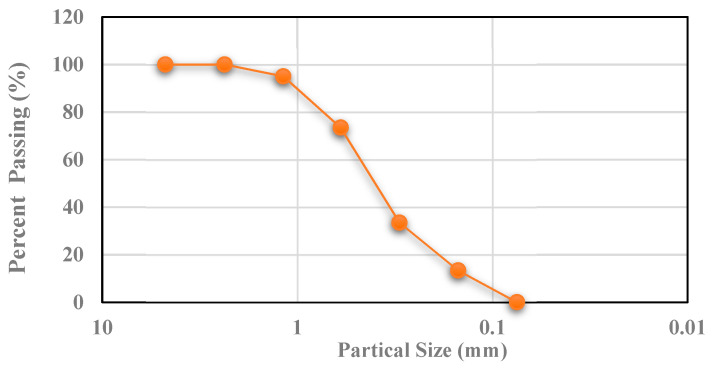
Particle size distribution of fine aggregate.

The first stage involved finalizing the design of the UHPC mixture and developing a new casting method using locally available materials for producing UHPC. The hardened mechanical properties, including compressive strength, were investigated for each of the trial mixes. In addition to the well-known aspect of the high compressive strength of UHPC, the focus was on achieving a flexural strength of about 30 MPa. Materials used for UHPC included cement, micro silica, fine aggregate, steel fibers, and superplasticizer. Ordinary Portland cement (OPC) Type I according to ASTM C 150 [37] with a specific gravity of 3.15 was used. The specific gravity and absorption values of fine aggregates were 2.56 and 0.4%, respectively. The particle size distribution of fine aggregate (FA) used in this paper is shown in Figure 1. The specific gravity and specific surface area of micro silica (MS) used in this study were 2.25 and 20,000 cm^2^/g, respectively. Table 1 shows the physical and chemical characteristics of raw materials used in this study. Copper-coated micro-steel fibers (straight steel-wire) used in this work were supplied through the local agency in Najran city, Saudi Arabia. The length, diameter, aspect ratio, and volume fraction of micro-steel fibers were 13 mm, 0.2 mm, 65, and 2%, respectively, with a maximum tensile strength of 2500 MPa. Table 2 presents the steel fiber specifications. A high-range water reducing agent (superplasticizer), Glenium^®^110M, was sourced from a local company (BASF), Najran, Saudi Arabia, of medium to dark brown colored liquid with a specific gravity of 1.065. The water to cementitious ratio of 0.15 was kept constant. The final mixture resulted in about 160 MPa compressive strength (2-inch cube) and flexural tensile strength of over 30 MPa. The materials used for the normal weight concrete (NC) include OPC, FA, coarse aggregate (CA) with a specific gravity of 2.65 and 1% absorption capacity, and tap water. Water to cement ratio of 0.42 was used to achieve a cylindrical compressive strength of about 40 MPa.

All materials, with the exception of the steel fibers, are locally available. The casting procedure of UHPC involves mixing the dry materials in a revolving mixing bowl, adding water and superplasticizer, and finally adding steel fibers. It should be noted that satisfactory production of UHPC requires a finely tuned preparation method. Table 3 shows the mixed design details of UHPC and NC used in the present study.

### 2.2. Preparation of the Test Specimens

**Figure 2:**
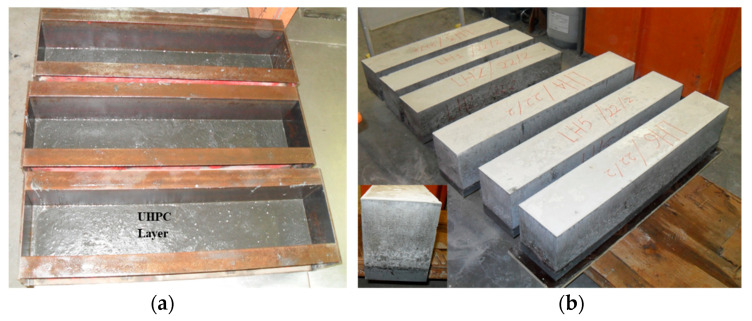
(**a**) UHPC layers prior casting the NC, (**b**) Hybrid NC-UHPC beams before testing.

The hybrid NC-UHPC beam was designed to fail in flexure, precluding shear failure. The beams did not have shear reinforcement. All specimens were made with the consistent UHPC mix, and the casting technique, the mix design, and the casting method were kept identical. The casting process of the composite NC-UHPC beam is shown in Figure 2. The details of hybrid NC-UHPC beam specimens used for the experimental program are presented in Table 4. The specimens were designed considering the influence of the UHPC thickness, which was considered a key variable. In addition, four different testing spans were also considered. A total of 24 specimens were cast, in the four groups, with four different thicknesses of UHPC layers 20, 40, 25, and 50 mm and different testing spans (630 mm, 750 mm, 900 mm, and 1100 mm) with two different cross-sections (150 mm × 150 mm and 150 mm × 200 mm). Only one curing regime was used, such that the bottom UHPC layer was the first cast into a mold, leaving the top surface unfinished, and allowed to self-cure for 2 days (Figure 2a) before placing the vibrated NC layer. The top face of all samples was trowel-finished. Figure 2b displays the hybrid beam specimens with a layer of UHPC at the bottom. All hybrid beam specimens had standard water curing for 28 days before testing.

### 2.3. Evaluation of Mechanical Properties of UHPC and NC

Compressive strength tests were performed as per ASTM C109 [38] for standard cubes and ASTM C 39 [39] for cylinders. UHPC concrete cubes were tested in a compression-testing instrument with a capacity of 3000 kN after 2 days of heat curing at 90 °C. Compression tests on cylinders of 75 mm × 150 mm were performed by means of a hydraulic compression machine of 2000 kN capacity with strain gauges applied on the specimen connected to the data logger to capture the strain and load results. The compressive strength of normal concrete was evaluated on 75 mm × 150 mm specimens as per ASTM C 39 [39] after 28 days of water curing.

Split cylinder tension tests were conducted on concrete prism 75 mm × 150 mm to evaluate the indirect tensile strength of UHPC specimens according to ASTM C496 [40]. The experiments were conducted using a compression testing machine of 2000 kN capacity. Direct tension test of UHPC was determined using dog bone-shaped specimens according to ASTM D638 [41] using a universal testing machine of 400 kN capacity. The dog bone specimens were instrumented using two strain gages connected to a data logger to collect load and strain data. Load tests on the specimens were translation-controlled with 0.1 mm/min constant loading velocity. The two ends of the specimen were fixed in the testing machine, and the geometry of the dog bone guaranteed that the cracks take place in the central zone. The arrangement of the setup was wisely tested before starting the test to avoid any eccentricity. The average tensile strain of the two strain gages was obtained. A standard four-point flexural loading test as per ASTM C 78 [42] was used to evaluate the flexural performance of UHPC and NC prisms. Flexural load tests were performed on 30 UHPC prisms with dimensions 40 mm × 40 mm × 160 mm after 48 h of heat curing using a universal testing machine with a constant loading velocity of 0.5 mm/min.

### 2.4. Testing Hybrid Beam Specimens

**Figure 3:**
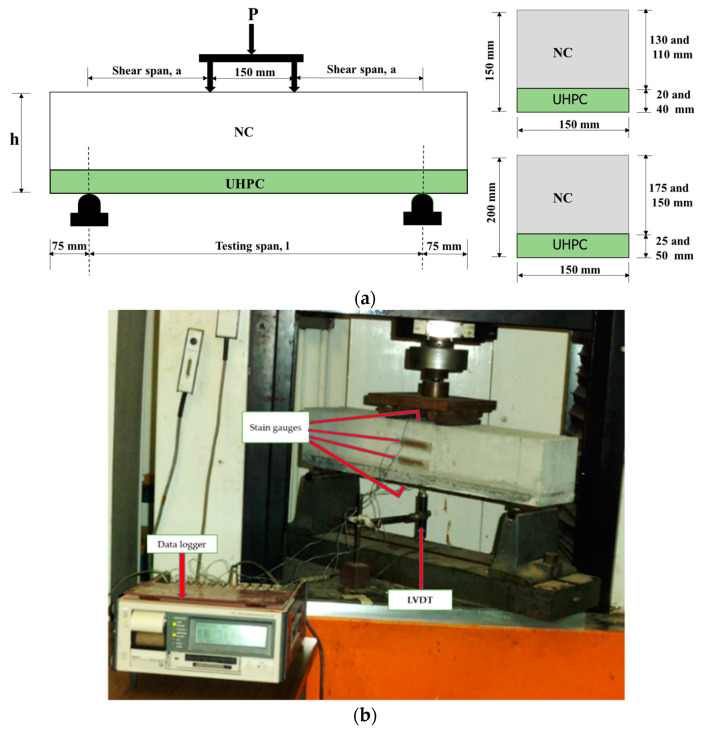
(**a**) Schematic for the load test of hybrid NC-UHPC beams; (**b**) hybrid test beams loaded under testing machine.

The hybrid NC-UHPC specimens were subjected to a four-point bending test under monotonically increasing load until failure using an Instron testing machine. Figure 3a shows a schematic for the load test, and Figure 3b shows the hybrid test beam loaded in the testing machine. The applied loads are spaced at a fixed distance of 150 mm (Figure 3a with varying shear spans for all hybrid NC-UHPC beams, as shown in Table 4. The ratio of shear span/depth (a/h) of all hybrid beams was larger than 1.5 to ensure a flexure mode of failure.

Deflection in the beam was measured with a linear voltage displacement transducer (LVDT) supplied by Tokyo Sokki Kenkyujo-Co., Ltd. (Tokyo, Japan). The LVDT was located at the bottom side of the beam in the central portion at mid-span (Figure 3b). Four strain gages were positioned at four different locations, on top and bottom surfaces and at 1/3 and 2/3 of the beam depth on the vertical side of the beams to observe the strain distribution across the depth (Figure 3b). Strain gauges of 60 mm gauge length type PL-60-11-3LJCT-F supplied by Tokyo Sokki were used in this study. All hybrid beam specimens were tested at a loading velocity of 0.5 mm/min. The deflection and strain values were recorded for every step of loading. The load–displacement path after the peak load was also recorded to observe softening behavior. In addition to the measurement of the strain and deflection, crack propagation, as well as the mode failure of all specimens, were also noted.

## 3. Experimental Results and Discussion

### 3.1. Results and Discussion for UHPC and NC Used in Hybrid Beams

#### 3.1.1. Compressive Strength of UHPC and NC

**Figure 4:**
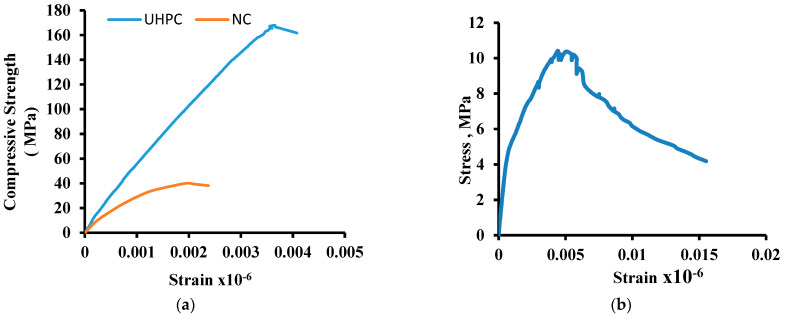
Stress–strain diagram: (**a**) UHPC and NC under compression, (**b**) UHPC under direct tension.

The average compressive strength of UHPC was obtained from three specimens of 75 mm dia. × 150 mm length cylindrical specimens were about 160 MPa with an average axial compressive strain of 0.0038. The average compressive strength of 50 mm size cubes from three specimens was 170 MPa with a standard deviation of ±8 MPa. A typical stress–strain diagram in compression obtained from load test on cylinders is shown in Figure 4a. The stain values were recorded using two strain gauges. A linear response can be observed up to nearly 120 MPa. From the compression stress–strain curves, the modulus of elasticity of UHPC was computed to be 55 GPa, which agrees with the values reported in previous studies [43,44,45,46,47]. The measured value of Poisson’s ratio was found to be about 0.22. Figure 4a also shows the stress–strain diagram for normal concrete (NC). The NC specimens perform elastically until the peak load, followed by a fast softening, loss of strength, and a sudden collapse in an explosive mode. The average results of the compressive strength and elastic modulus of three specimens were 40 MPa and 30,000 MPa, respectively.

#### 3.1.2. Split and Direct Tensile Strength of UHPC

The indirect split tensile tests result for UHPC was measured to be 26 MPa with a standard deviation of ±1.33 MPa. This value of split tensile is around five times higher compared to the normal concrete. Graybeal et al. [47] also reported similar results of 25 MPa under steam curing regimes. Tensile strength values as high as 30 MPa were also reported in the references [48,49,50,51]. The UHPC cylinders exhibit longitudinal cracking at failure without splitting in half due to the bridging influence of small size steel fibers, in addition to the dense microstructure of UHPC [52,53,54].

The average direct tensile strength of three UHPC specimens obtained on dog bone-shaped specimens was 10 MPa with a standard deviation of ±1.0 MPa. A typical stress–strain response of the prism tested under direct tension is shown in Figure 4b. The behavior of UHPC under direct tension is described by nearly linear stress increase up to the initiation of the first crack, after that limited strain hardening until the maximum strength was achieved, followed by a softening portion until failure.

#### 3.1.3. Flexural Strength of UHPC

**Figure 5:**
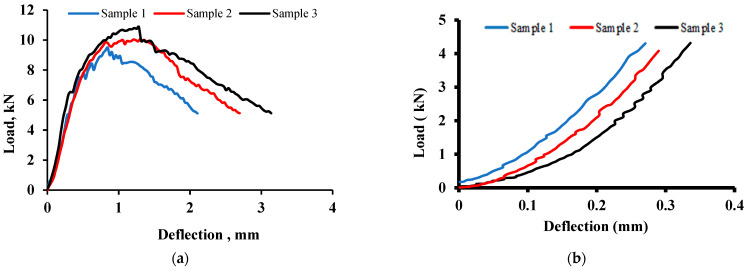
Typical load–deflection plot for (**a**) UHPC prisms under flexure and (**b**) UHPC prisms without steel fibers.

The UHPC prisms were tested for flexure under four-point loads after 48 h of heat treatment at 90 °C in the oven. The average flexural strength of three specimens was about 27 MPa with a standard deviation of ±2 MPa. Figure 5a shows a typical load–deflection plot of three identical specimens. The flexural behavior of the UHPC specimens is described by a linear elastic portion prior to cracking, followed by the presence of many fine cracks, a limited strain-hardening phase until the maximum strength is reached and finally, a gradual softening mode associated with the widening of the crack.

As shown recently [51,52,53], the orientation and distribution of fibers in UHPC can have a significant influence on mechanical performance. This effect must be reflected in structural uses where the difference in fiber distribution in bigger sections can lead to a significant variation in the mechanical behavior of concrete. The casting direction of fresh UHPC is predicted to affect the orientation of the fiber, and when workability is excessively high, or there is more vibration on the specimens during casting, the steel fibers may also settle down, leading to disturbed orientation and dispersion of steel fibers [52]. Fiber orientation appears to have a very large effect on the flexural strength of the tested prisms. The aspect ratio of fibers also plays an important role in flexural performance, as reported by many researchers [53,54]. It is well known that the size influence is not revealed in the flexural test specimens since all prisms had the same size and length. It is later seen that the flexural tensile strength of UHPC does really vary significantly, with the strength being significantly lower for larger sizes.

In order to determine the flexural capacity of the cementitious matrix without steel fibers, three small prisms were made of UHPC with no steel fibers in the mix and examined in a four-point bending test procedure according to ASTM. The load–displacement diagram of three identical specimens tested under flexural loading with no steel fibers is shown in Figure 5b. As expected, the flexural behavior was very brittle without any ductility. The average flexural strength of 13 MPa was obtained, which is about 50% of the strength of UHPC specimens with steel fibers. All three specimens exhibited sudden failure, lacking any softening mode after peak load.

#### 3.1.4. Key Mechanical Properties of UHPC and NC

The average results of all tests conducted on UHPC and NC are listed in Table 5.

### 3.2. Results and Discussion of Hybrid NC-UHPC Beams

#### 3.2.1. Flexural Load Test Results of Hybrid NC-UHPC Beams

**Figure 6:**
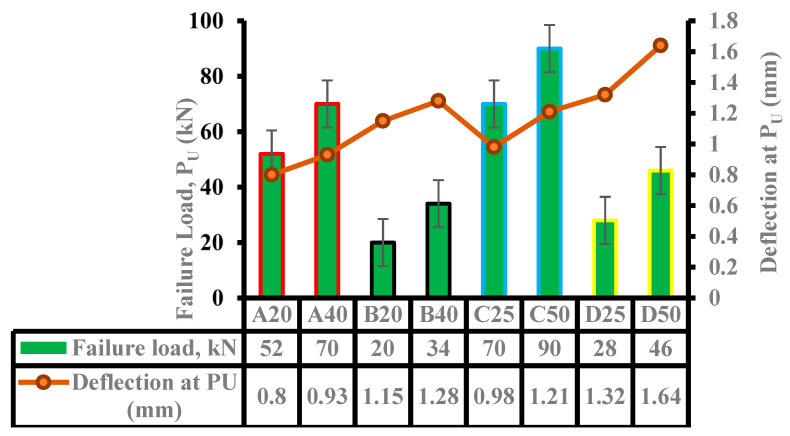
Average failure load and deflection of hybrid beams.

Flexural load tests were conducted on hybrid NC-UHPC with four different spans (Groups A to D) ranging from 630 mm to 1100 mm, and each span having two different UHPC thicknesses. Groups A and B have a thickness of 20 mm and 40 mm, whereas Groups C and D have thicknesses of 25 mm and 50 mm. The details of spans, failure load, and moment capacity of three identical specimens are shown in Table 6. The average results of three specimens for maximum flexural failure load, P_U_, and the corresponding deflection recorded at mid-span for all hybrid beam specimens are presented diagrammatically in Figure 6.

It can be seen from Figure 6 that for each of the four (4) spans, the failure load increases when the thickness of the UHPC layer is doubled. For spans of 630 mm and 750 mm, the failure load increases by 34.6% and 28.6%, respectively, when the thickness is doubled. For longer spans of 900 mm and 1100 mm, the failure load increases by 70% and 64.2%, respectively, as the thickness is doubled. The failure load decreases by 61.5% for specimens A20 and B20, as the spans increase from 630 mm to 900 mm, whereas for the 40 mm thick UHPC layer, it decreases by 51.4%. For 25 mm and 50 mm thick UHPC layers, the reductions are 60% and 49%, respectively, as the span increases from 750 mm to 1100 mm. It can be observed from Figure 6 that the deflection increases by 16.3% and 11.3% for 630 mm and 900 mm spans as the thickness are doubled, whereas it increases by 23.5% and 24.2% for 750 mm and 1100 mm spans as the thickness is increased from 25 mm to 50 mm. For the same shear span (a) to UHPC thickness (t) ratio of a/t = 12 (specimens A20, C25), the moment capacity increases by 68%, showing an increase in UHPC thickness by 5 mm results in a significant increase in the flexural capacity. For a/t = 6 (specimens A40, C50), the increase in the capacity is about 60.7%. Similarly, for a/t = 9.5 (B40, D50) and a/t = 19 (B20, C25), the moment capacity increases by 43.6% and 41.6%, respectively. With respect to the ratio of shear span and total depth (a/h) of the composite beam, the moment capacity increases significantly as the UHPC layer thickness is doubled. For example, for a/h = 1.6, the capacity increases by 34.6%, whereas, for a/h = 2.5, it increases by 70% as the thickness of UHPC is increased from 20 mm to 40 mm. When the total depth of the composite beam is increased from 150 mm to 200 mm, and the UHPC thickness of 25 mm is doubled, the moment capacity increases by 28.6% and 64.2% for a/h of 1.5 and 2.4, respectively.

#### 3.2.2. Mode of Failure of the Hybrid NC-UHPC Beams

**Figure 7:**
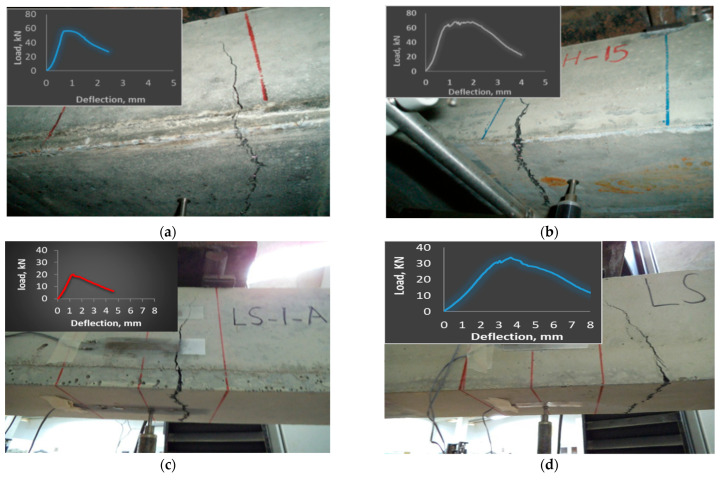

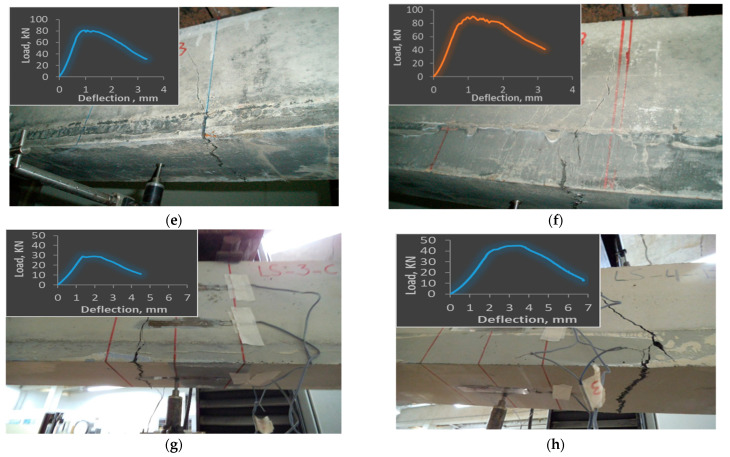
Cracking and typical load–deflection response of hybrid beams. (**a**) A20; (**b**) A40; (**c**) B20; (**d**) B40; (**e**) C25; (**f**) C50; (**g**) D25; (**h**) D50.

The failure modes of the eight hybrid NC-UHPC beams (Groups A to D) of varying thicknesses, along with their load–deflection responses, are shown in Figure 7. 

The failure is, in general, characterized by a single vertical crack traversing across the UHPC layer into the NC layer, reaching almost to the top of the beam. In some specimens, several fine cracks appear before a single wide crack takes control. Cracks in all specimens extend across the width of the beam. The cracks in all specimens are formed either between the loading points or adjacent to the loads a little distance outside. Figure 7a,b show the failure mode of specimens A20 and A40 of 630 mm span. The cracks commencing from the bottom of the beams propagate vertically across the NC-UHPC interface into the NC layer extending to the top of the beam. The width of the crack for the A40 specimen with a thicker UHPC layer is wider as compared to the A20 specimens. A significantly higher energy dissipation can be observed in the A40 specimen. The load–deflection curve shows a strain-hardening portion significantly greater than the A20 specimen. The influence of steel fibers on the bridging mechanism and uncracked portion of the UHPC layer is evident, which offers post-cracking ductility to the beam. The effect of the a/t ratio on the failure mode can be seen with A20 (a/t = 12), showing lower ductility and energy dissipation. The failure mode of the specimen B20 (a/t = 18.8) and B40 (a/t = 9.4) are shown in Figure 7c,d. The failure is again characterized by a single, wide crack formed within the load (B20 specimen) and outside the load point for the B40 specimen. Failure is characterized by a significantly lower strain-hardening region compared to A-type specimens. The uncracked portion within the load point provides higher energy dissipation in B40 as compared to the B20 specimen.

The failure mode of the UHPC-hybrid beam in Group C with 25 mm and 50 mm thick UHPC layer is shown in Figure 7e,f. In 50 mm thick specimen C50, several hairline cracks are developed at the bottom side, extending only in the UHPC layer between and outside the loading points when the applied load is close to the failure load P_U_. A small hardening portion during which the cracks grow wider, moving upward into the NC layer, can be observed in the specimen. At peak load, a straight upward single vertical crack develops outside the left loading point, which extends well into the NC layer. This is followed by a moderate softening behavior up to failure, with crack width increasing as the bridging effect of steel fiber vanishes. In contrast, for 25 mm thick UHPC layer specimen C25, the flexural mode of failure illustrates the development of a single crack between the loading points at peak loading. The crack width increases over a small strain-hardening region, after which the softening ensues as the load decreases with increasing crack width. The crack extends across the NC-UHPC interface deep into the NC layer over the width of the beam.

In the specimens of Group-D, with a/t ratio of 19 (D25) and 9.5 (D50) and a beam span of 1100 mm, the flexural failure mode is almost identical to the Group-C specimen (Figure 7g,h), with lower failure loads. A single vertical crack within the loading point (D25) and outside the right loading point (D50) extending across the width is observed. For all specimens tested, the UHPC layer imparts significant ductility to the hybrid NC-UHPC beam and functions as a reinforcement in the normal beam. A well-developed softening curve can be seen after the ultimate load with the fibers bridging the crack, imparting resistance to crack development. Once the fibers at the bottom of the UHPC layer are pulled out as the crack width increases, the steel fiber in the upper layer swings into action, resisting the load. The failure was seen to occur once most of the fibers lost their resistance capacity.

#### 3.2.3. Load–Deflection Response of the Hybrid NC-UHPC Beams

**Figure 8:**
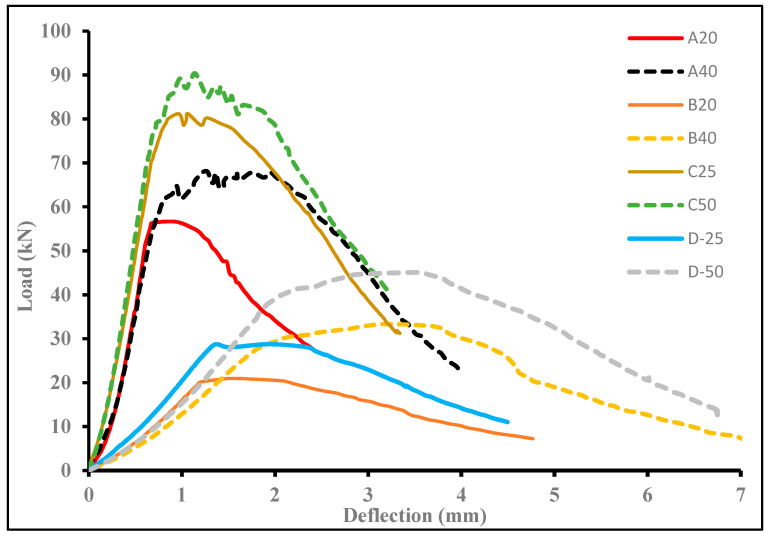
Load–deflection curve for all hybrid beams under flexural loading.

Figure 8 shows the combined load–displacement response of all eight-hybrid beams with two different depths, 150 mm and 200 mm, and four different thicknesses of UHPC layers. The moment capacity increases as the a/h ratio of the beam decreases. The effect of doubling the thickness of the UHPC layer in enhancing the moment capacity increases significantly from 28.6% for a/h = 1.5 to 70% for a/h = 2.4. The thickness of the UHPC layer enhances the ductility of the hybrid beams. When the thickness of the UHPC layer increases from 25 mm to 50 mm for Groups C and D, Group D specimens with a/h = 2.4 show a significant increase in ductility. Whereas, for a/h = 1.5, the moment capacity increases but with a slight change in the ductility. For Group-A specimens for a/h = 1.6, the ductility increase is significant with enhanced moment capacity, and so do the Group-B specimens.

The flexural behavior of the Composite NC-UHPC beams is characterized by a linear response up to the point of cracking. For higher thicknesses of the UHPC layer, the ultimate load, in general, is higher than the cracking load (Refer to Table 6). The larger thickness with a higher volume of steel fibers provides resistance to the propagation of cracks across the depth of the UHPC layer. For specimen C50 the ultimate load is 22% higher than the cracking load, whereas, for the D50 specimen, a large strain hardening region can be seen up to the ultimate load, with displacement increasing from about 2 mm at cracking load to about 3.5 mm at ultimate load. The ultimate load is 17% higher than the cracking load for D50. The A40 and B40 specimens, on the other hand, have an ultimate load higher by about 27% and 9%, respectively, with the B40 specimen showing significant displacement up to the achievement of the P_U_ value. For specimens B20 and D25, the cracking and ultimate failure load are the same as seen in Table 6. The load–deformation for these specimens follows closely, with a significant strain hardening portion and higher ductility. For specimens A20 and C25 with similar a/h ratio, the ultimate load is higher than the cracking load by 13% and 9%, respectively. Both specimens show similar load–deflection responses, with C25 having significantly higher moment capacity (68%) compared to A20.

After the peak load, a horizontal portion with varying lengths can be seen in Figure 8. All specimens after the peak load show a softening curve up to failure. This indicates a non-brittle failure in the NC-UHPC hybrid beams. All failures, as mentioned in the previous section, are characterized by a single vertical crack within or adjacent to the loading points. Due to the significant difference between the cracking load (uncracked section) and ultimate load (cracked section), the deflection in the beam could be computed using the cracked moment of inertia. This approach is adopted in the section that follows. It is also evident from the Figure 8, that the load–deflection behavior of specimens A20 and C25, A40 and C50 (a/h = 1.6 and 1.5), B20 and D25, B40 and D50 (a/h = 2.5 and 2.4) follow closely.

The flexural capacity of hybrid beams can vary due to size, for example, the width of the beam. In larger size specimens, the effect of dispersion and orientation of steel fibers in UHPC is more pronounced. Magureanu et al. [55] reported the results of flexural load testing of UHPC beams of two different sizes (40 mm × 40 mm × 160 mm and 100 mm × 100 mm × 300 mm) and concluded that the flexural tensile strength of small size beams was about 1.47 higher than the larger one. Moreover, Duy Liem et al. [56] agreed with the factor of 1.47 by investigating the size effect by testing flexure under 4-point bending in three different sizes of UHPC specimens.

**Figure 9:**
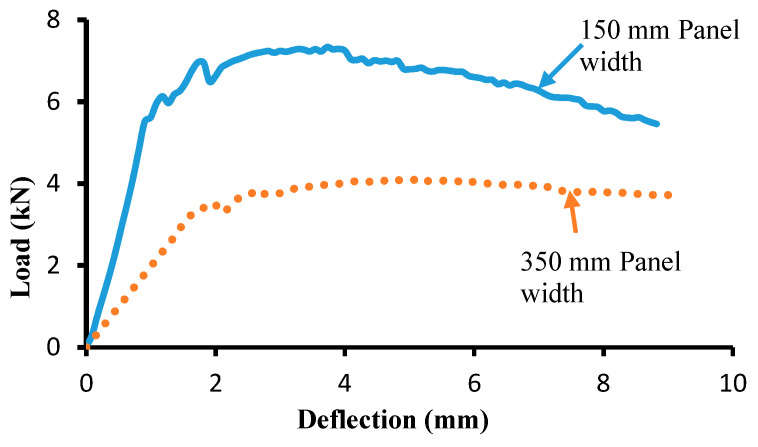
Effect of UHPC panels width on the flexural strength.

In order to confirm the findings related to the influence of specimen size on the flexural capacity of beams with UHPC, panels of two sizes, 600 mm × 30 mm × 150 mm and 600 mm × 30 mm × 350 mm (height × depth × width), were cast and subjected to four-point flexural load over a clear span of 500 mm. The load–deflection response of the UHPC beams is shown in Figure 9. The average peak load of three specimens for a 150 mm wide specimen is 7.2 kN, which is reduced to about 4 kN for a 350 mm wide specimen. Both specimens show significant ductility after the peak load. The load capacity decreases by about 45% when the width of the UHPC beam is increased.

#### 3.2.4. Strain Distribution across the Depth of the Beam

Strain developed in the NC-UHPC hybrid beams was measured by strain gauges attached at the top and bottom and two gauges attached on the sides of the beams, as shown in Figure 3b. The strain gauges were attached at the mid-span of the hybrid beam. The measured strains are used to find the depth of the neutral axis of these beams. Data for two sets of hybrid beams specimens, A20 and B20, of the same thickness of UHPC layer and different spans of the beams and D25 and D50 with of different thickness and same span are discussed here for brevity (other data are available with authors). The recorded values of strains at four different locations and at different levels of load are shown in Table 7 for A20 and B20 and Table 8 for D25 and D50. The measured and computed depth of the neutral axis for the four hybrid beam specimens are also shown in the tables.

**Figure 10:**
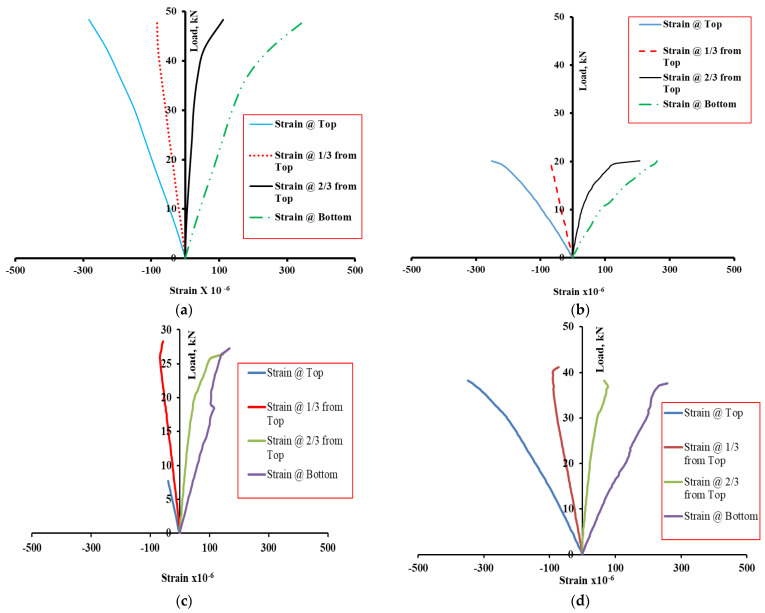
Load–strain curve: (**a**) beam A20, (**b**) beam B20, (**c)** D25, and (**d**) D50.

The load–strain curves for the beams A20, B20, D25 and D50 at 90% of P_U_ are shown in Figure 10 for the strains measured at the top, bottom, 1/3rd, and 2/3rd of the depth from the top. For specimen A20, the load–strain curve is shown in Figure 10a. The tensile strains in gauges at the bottom and 2/3rd depth are linear initially, up to 70% of the P_U_ for bottom gauges and 80% of the P_U_ for the gauge at 2/3rd depth. The strains become nonlinear beyond these load levels. The compressive strains at 1/3rd depth are linear up to failure; however, the top strain shows slight nonlinearity beyond 80% of P_U_. On the other hand, for specimen B20 (Figure 10b), the tensile strain becomes nonlinear at about 50% of P_U_ at the bottom face. The compressive strain at the top is almost nonlinear except at nearly 95% of P_U_. Similar observations can be made for the beams D25 and D50 (Figure 10c,d). The load–strain response is almost linear, with the strain at the top face showing slight nonlinearity at higher load. At lower load levels (service load), the strains in the hybrid beam are linear. The linearity of the strain confirms that no slip occurs between the UHPC and the normal concrete and adequate bonding exists between NC and UHPC layers at the interface.

**Figure 11:**
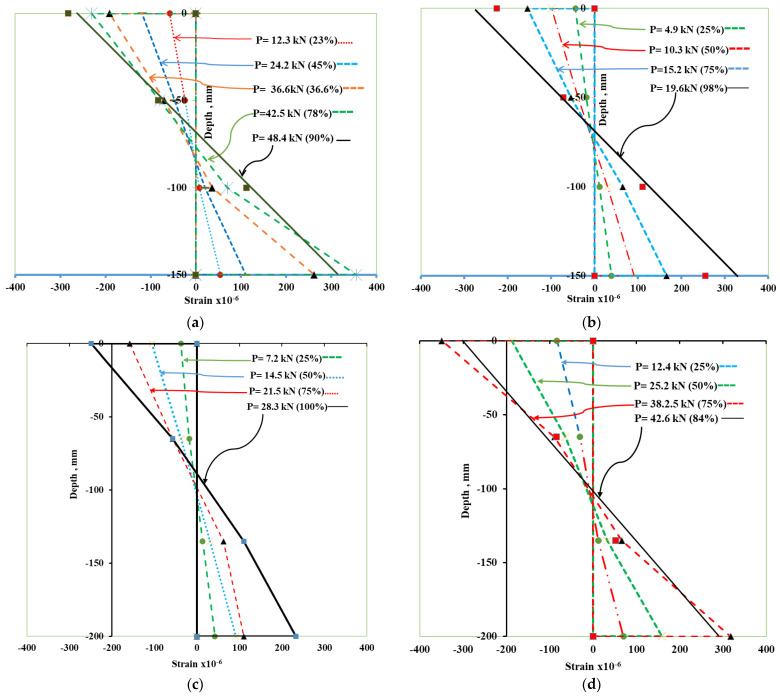
Strain diagram through the depth of hybrid beam: (**a**) beam A20, (**b**) beam B20, (**c**) beam D25, and (**d**) Beam D50.

The strain diagram across the depth of NC-UHPC hybrid beams obtained from measured values of strains at four locations are shown in Figure 11a,b for the beam specimens A20 and B20 and Figure 11c,d for the beam specimens D25 and D50. The variation in strains for five increasing load levels for specimen A20 and four load levels for other specimens are shown in the figures. These load levels represent strains at lower levels of load and close to failure. For all specimens, the compressive strains for lower load levels are almost linear. For specimen B20, the tensile strains follow the linear path without any divergence. However, for specimens A20, D25 and D50, the slope of the line change below the neutral axis at the NC-UHPC interface at the load level equal to 75% of P_U_ and higher level. This divergence can be attributed to a possible slip at the interface. It can also be seen from Figure 11 that the neutral axis moves upward as the load level increases. This movement is in the vicinity of 5–8 mm.

### 3.3. Analytical Computation of Stresses in Hybrid NC-UHPC Beams

#### 3.3.1. Neutral Axis and Moment of Inertia of Hybrid NC-UHPC Beams

The flexural capacity of NC-UHPC beams can be computed based on the flexural theory of beams. The following assumptions are made for the analytical NC-UHPC model:

1. The beam section remains plane under loads;
2. The beam bonding takes place without any slip at the NC-UHPC interface, i.e., there is a perfect bond;
3. Properties of NC-UHPC in the hybrid beam are expressed by material constitutive law.

**Figure 12:**
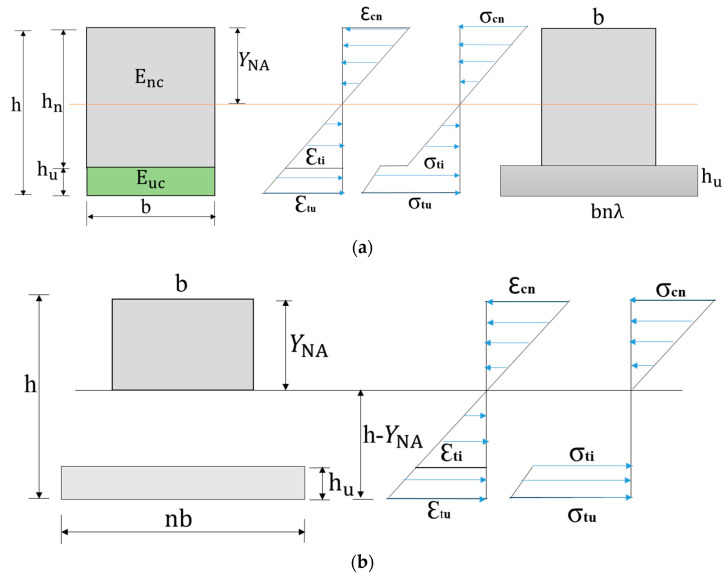
Moment of inertia and stresses in hybrid beam: (**a**) Uncracked section (λ = 1) and partially cracked UHPC layer (**b**) cracked NC layer.

From the experimental program conducted, it was observed that cracking in the beams may commence either at the bottom of the UHPC layer or at the bottom of the NC layer. No shear cracking was observed at the interface of NC-UHPC, which was reported in [34]. The computation of the neutral axis and the moment of inertia (MI) of the hybrid sections for the computation of stresses depends on the state of cracking. Just prior to the development of cracking either in the NC or the UHPC layer, the uncracked MI (I_uc_) can be computed in which both NC and UHPC are effective (Figure 12a). If the cracks develop in the NC layer and the UHPC layer is uncracked, then MI is (I_crn_) in which NC is cracked below the NA (Figure 12b). There could be partial cracking in the NC layer, and a portion of the section below the NA may be active in resisting the tension. If the cracks initiate at the bottom of the UHPC layer and the NC is uncracked, then the MI (I_cru_) can be determined. The steel fibers in the UHPC resist the cracks, and the UHPC layer is not fully effective. The stress diagram is shown in Figure 12a. A simplified approach in which a reduction factor λ may be applied while transforming the UHPC layer to NC for computing the NA and MI. The factor accounts for cracking in the UHPC layer.

For the uncracked section and for the case where the crack initiates in the UHPC layer, the depth of the neutral axis, Y_NA_ measured from the top of the section, and the uncracked moment of inertia (I_uc_) and cracked UHPC MI (I_cru_) of the composite beam was calculated from Equations (1) and (2). For the uncracked section λ = 1, and for the UHPC cracked section, the value of λ depends on the extent of cracking in the UHPC layer. The flexural strength of the composite beams can be determined using the appropriate values of depth of NA and the moment of inertia.

(1) $$Y_{\mathrm{NA}}=\frac{h_{n}{}^{2}+2{\lambda \mathrm{nh}}_{u}h-{\lambda h}_{u}{}^{2}}{2\left[h_{n}+{\lambda h}_{u}\right]}$$

(2) $$I_{\mathrm{uc}/\mathrm{cru}}=\frac{1}{3}{\mathrm{bY}}_{\mathrm{NA}}{}^{3}+\frac{1}{3}b{\left(h_{n}-Y_{\mathrm{NA}}\right)}^{3}+{\mathrm{bn}\lambda h}_{u}\left[\frac{h_{u}{}^{2}}{12}+{\left(h-Y_{\mathrm{NA}}-\frac{h_{u}}{2}\right)}^{2}\right]$$

For the cracked normal concrete section (Figure 12b), Y_NA_ was measured from the top, the NA was calculated by solving Equation (3), and the cracked moment of inertia (I_crn_) was calculated from Equation (4). Partial cracking in the UHPC layer can be accounted for by the factor λ.

(3) $$\frac{1}{2}Y_{\mathrm{NA}}{}^{2}={n\lambda h}_{u}\left(h-Y_{\mathrm{NA}}-\frac{h_{u}}{2}\right)$$

(4) $$I_{\mathrm{crn}}=\frac{1}{3}{\mathrm{bY}}_{\mathrm{NA}}{}^{3}+\frac{1}{12}{n\lambda \mathrm{bh}}_{u}{}^{3}+{\mathrm{nb}\lambda h}_{u}{\left(h-Y_{\mathrm{NA}}-\frac{h_{u}}{2}\right)}^{2}$$

The values of elastic modulus for UHPC and NC, as presented earlier in Table 5, were 55 GPa and 30 GPa, respectively. The modular ratio (n = E_uc_/E_nc_) of the hybrid section is 1.833. The computed values of NA and MI for the uncracked, cracked NC layer and cracked UHPC layer of hybrid beam sections (Figure 12) are displayed in Table 9. For the cracked UHPC layer, the value of λ = 0.15 is assumed.

#### 3.3.2. Depth of Neutral Axis

**Figure 13:**
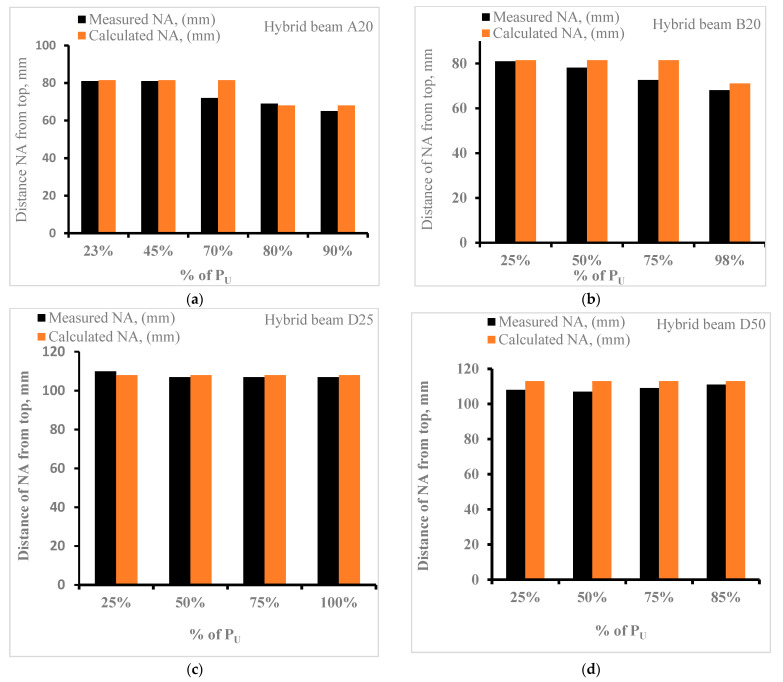
Measured and calculated distance of neutral axis (X) from top: (**a**) beam A20, (**b**) beam B20, (**c**) beam D25, and (**d**) beam D50.

The strain variation along the depth (Figure 11) shows that the neutral axis moves slightly upward at a higher load level. This movement is in the vicinity of 5–8 mm. Figure 13 shows a comparison between the computed and average measured depth of the neutral axis in the beam specimens A20 (Figure 13a) and B20 (Figure 13b). For specimen A20, at a load level of 23% and 45% of P_U_, the values are identical; however, at higher levels (70%, 80%, 90%), the usage of I_cru_ for computation of depth of neutral axis is slightly higher compared to the measured values. The UHPC layer may be marginally stiffer than assumed in the computations. For specimen B20, at load levels up to 25%, the depth of the neutral axis is also identical, but for 50%, 75%, and 98%, the calculated values are marginally higher. For specimen D25, the measured and calculated values of NA are almost the same at all load levels; however, for specimen D50, the calculated values are slightly higher at all load levels. For service load levels, say up to 30% of P_U_, the neutral axis can be computed using an uncracked section.

#### 3.3.3. Cracking Load for the Hybrid Beams

(5) $$P_{\mathrm{cr}.}=\frac{2{\;f}_{r}{\;I}_{\mathrm{uc}}}{{a\;Y}_{\mathrm{NA}}}\;\mathrm{where}\;M_{\mathrm{cr}}=\frac{f_{r}{\;I}_{\mathrm{uc}}}{{\;Y}_{\mathrm{NA}}}$$

where f_r_ is the flexural strength, I_uc_ is the uncracked transformed section (Table 9), a is the shear span, $Y_{\mathrm{NA}}$ is the bottom distance of neutral axis (N.A).

**Figure 14:**
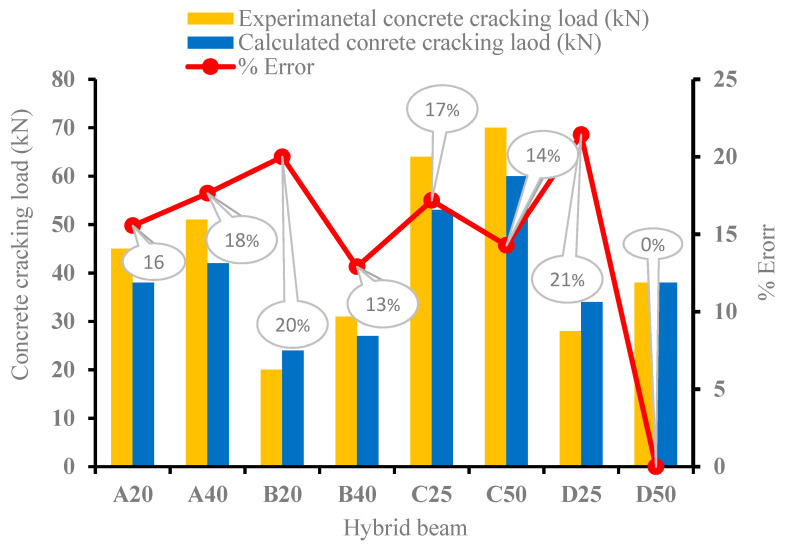
Experimental versus the calculated cracking load (P_cr_.).

The calculated cracking load and the average experimental cracking load for all tested beams are shown in Figure 14. The modulus of rupture for NC is taken as 6 MPa. The error ranges from 3% to 20%, with calculated values being lower in most of the cases except B20 and D25.

**Figure 15:**
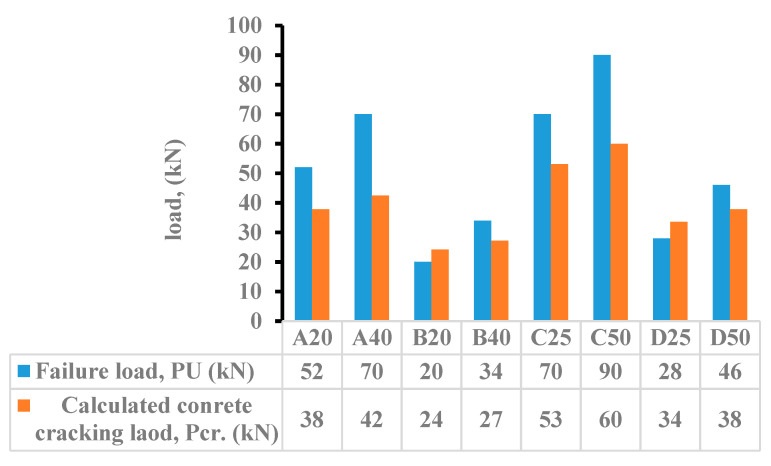
Experimental failure load (P_U_) versus the calculated cracking load (P_cr_.).

The experimental failure load (P_U_) and the calculated cracking load (P_cr_.) is shown in Figure 15. For specimen A20, the difference between the average ultimate load (52 kN) and the average cracking load (38 kN) is higher. The specimen cracks at the lower load level, and there is a transition from uncracked to the cracked concrete section. For specimen B20, the average cracking load (20 kN) and the average ultimate load (20 kN) are very close, and the section remains uncracked up to a high load and fails at a slightly higher load. For a/h ratio of 1.5 and 1.6, the cracking occurs at a lower load and increases significantly by values ranging from 32% to 36.8% for the smaller thickness of the UHPC and 52.5% to 66.6% when the thickness is doubled. For a higher a/h ratio of 2.4 and 2.5, the difference between the cracking and the ultimate load is in the range of 14% to 26%.

#### 3.3.4. Computation of Moment Capacity and Stresses

The moment capacity of the hybrid beam at experimentally measured and computed cracking load is shown in Table 10. For the purpose of comparison, the moment capacity of the NC beams of the same dimensions, unreinforced and reinforced, are also presented. Unreinforced NC beams have an ultimate moment capacity of 3.4 kN·m for the beam cross-section of 150 mm × 150 mm (beams A20, A40, B20, B40) and 6 kN·m for beam cross-section of 150 mm × 200 mm (beams C25, C50, D25, D50). For the NC beams reinforced with 3–8 mm diameter steel bars, the moment capacity ranges from 7.5 kN·m for the beam cross-section of 150 mm × 150 mm (beams A20, A40, B20, B40) to 10.7 kN·m for beam cross-section of 150 mm × 200 mm (beams C25, C50, D25, D50). The experimental results show that the NC-UHPC beams have moment capacity ranging between 3.8 kN·m and 6.1 kN·m for the beams A20, A40, B20, and B40, and between 6.7 kN·m and 10.5 kN·m for beams C25, C50, D25, and D50. The moment capacity is of the same order as the reinforced NC beam and significantly higher than plain concrete beams. The potential of using a UHPC layer as a replacement for steel is exhibited; however, verifications by tests on large-scale beams are warranted.

**Figure 16:**
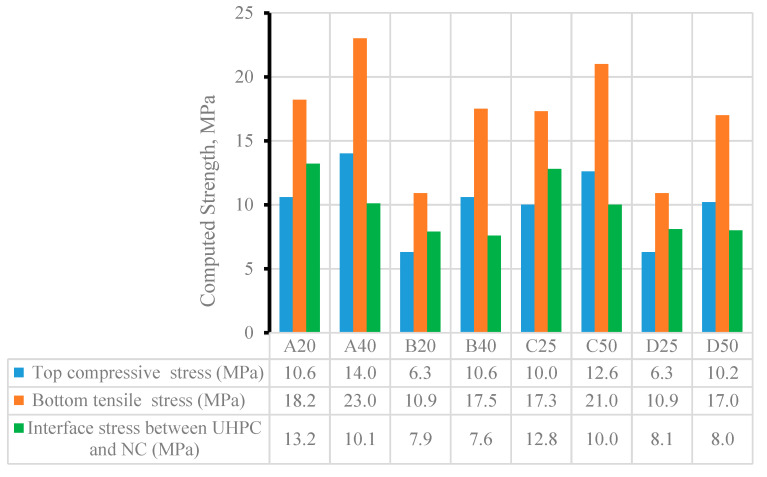
Experimental stresses in UHPC layer, at interface, bottom, and top of the hybrid beams.

Figure 16 shows the stress in the hybrid NC-UHPC beams, computed at measured cracking load using the NA and MI computed earlier. The tensile stress in the UHPC layer varies between 17.0 MPa and 23.0 MPa (at the bottom) and 7.6 MPa and 13.2 MPa (at the interface). The compressive stress in the NC layer varies between 6.3 and 14 MPa. The tensile stress in the UHPC layer increases when the thickness is doubled. For Groups A and C, with an a/h ratio of about 1.5, the stress in UHPC at the bottom increases by 21 and 26%, whereas for Groups B and D (a/h = 2.4), it increases by 56 and 60%. The compressive stress also showed a similar enhancement when the thickness of the UHPC layer doubled.

#### 3.3.5. Comparison of Measured and Calculated Stresses

**Figure 17:**
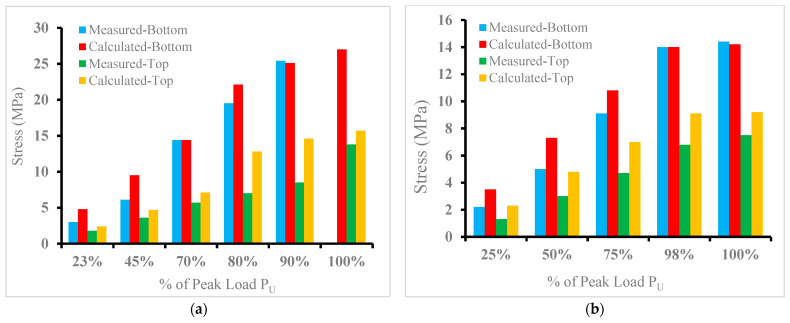

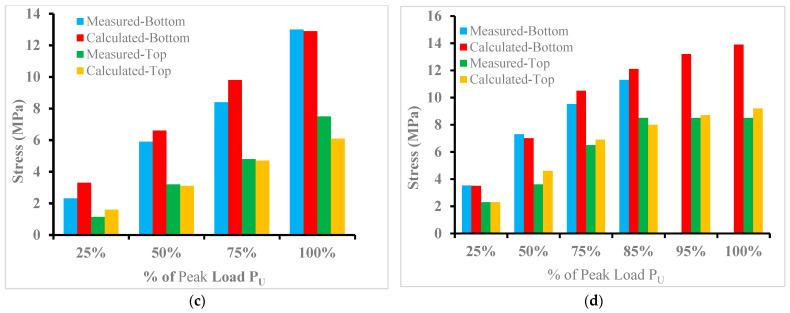
Measured and calculated stresses for selected load levels: (**a**) beam A20, (**b**) beam B20, (**c**) beam D25, and (**d**) Beam D50.

In general, the hybrid beam section is considered a cracked section as soon as the tensile strength of the UHPC layer at its bottom face goes beyond the computed tensile strength of the NC, as discussed in the previous sections. The comparison between the theoretical results calculated using a simple bending equation with the measured stresses from strains for the specimens A20, B20, D25, and D50 are presented in Figure 17. The flexural tensile stress at the bottom side of the hybrid beam was calculated with an elastic modulus of UHPC (E_uc_) of 55 GPa, while the compressive strain at the upper layer of NC was changed to stress by using the modulus of elasticity of NC (E_nc_) of 30 GPa (Table 5). At a smaller level of load, P < P_cr_., the stresses in the hybrid beam were evaluated by using an uncracked beam section, whereas the cracked beam section was used for computing the stresses at load level, P > P_cr_. (Table 6). Figure 17 shows a comparison of the calculated and measured stresses at the top and bottom of hybrid beams for different load levels up to 100% of failure load for the selected hybrid beams. The figure also demonstrated that, at all levels of load, the computed stresses are reasonably close to the stresses from the measured strains values, with the exception of some differences close to the ultimate failure load due to the nonlinearity that occurs close to failure load.

#### 3.3.6. Comparison of Measured and Calculated Deflection in Beams

**Figure 18:**
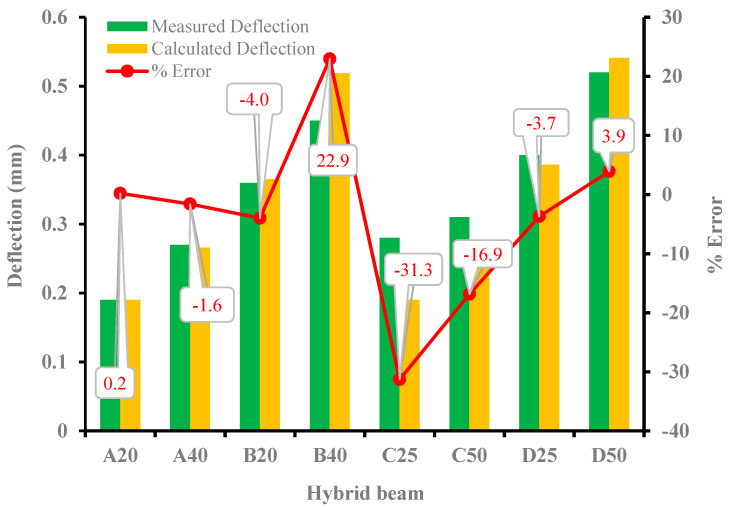
Comparison of measured and calculated deflection at cracking.

Figure 18 shows the average results of measured and computed deflections at the mid-span of all hybrid beams up to the concrete cracking load P_cr_. By using a simple deflection equation under flexural loading, as shown in Equation (6), the calculated deflections were obtained.

(6) $$\delta_{\mathrm{cr}}=\frac{P_{\mathrm{cr}.}a}{48{\mathrm{EI}}_{\mathrm{cr}}}\left(3L^{2}-4a^{2}\right)$$

where $\delta_{\mathrm{cr}}$ is the deflection at the center of the hybrid beam $E_{\mathrm{nc}}$ is the elastic modulus of NC, I is the moment of inertia of the section, $P_{\mathrm{cr}.}$ is the applied load, L is the span of the beam, and a is the shear span. The MI could be either the uncracked MI ($I_{\mathrm{uc}}$) just prior to cracking, MI with a portion of the UHPC layer cracked ($I_{\mathrm{cru}}$) and MI in which the NC below the neutral axis is cracked ($I_{\mathrm{crn}}$). Table 11 shows the measured and calculated deflection with various MI. The UHPC cracked MI assumes that 70% of the UHPC layer is effective due to cracking in the UHPC layer.

For all beams investigated, there is a big difference between the computed and the measured deflection of the eight hybrid beams when an uncracked section prior to cracking with a MI (I_un_) is used to compute deflections. The deflections are consistently lower, ranging from 39% to 144%. Similarly, if it is assumed that NC below NA is cracked and the MI (I_crn_) is used, the deflection is still under-predicted, with errors ranging from 37% to 142%. In most of the beams cracking was observed to initiate in the UHPC layer. A more appropriate equation for computation of deflection, with values closer to experimentally observed deflections, is based on partial cracking in the UHPC layer, as shown in Table 11. The difference between measured and computed deflection using (I_cru_) ranges from 0.2% to 31%. The UHPC layer is assumed to be cracked significantly and with a λ = 0.15 to match the measured deflection. Figure 18 shows the measured and calculated deflections based on a partially cracked UHPC layer.

## 4. Conclusions

An investigation was conducted to develop hybrid NC-UHPC concrete flexural members that utilize the ultrahigh tensile strength of UHPC and eliminate the need for passive steel reinforcement. The concept was explored by utilizing a thin layer of UHPC at the bottom side (tension side) of an NC layer. This experimental study reveals the feasibility of such a design; however, further studies are warranted. The following conclusions are drawn:

1. The experimental results on small-scale specimens show that hybrid NC-UHPC sections with a thin layer of UHPC in the tension zone below the thicker NC layer can be adopted for simple span beam/one-way slab-type members to carry flexural loads without using passive steel reinforcement. The thickness of UHPC layers should preferably be conserved at a proper depth since a greater thickness is not very efficient structurally as a load-carrying part. Steel reinforcement-free concrete beams and slabs can eliminate corrosion-related durability problems;
2. The mode of failure of the tested beams showed cracking in the UHPC layer predominantly with a single wide crack within the middle third. Additionally, the UHPC layer in the tension zone imparts a high ductility to the beam and significantly enhances the cracking resistance and moment capacity due to the presence of steel fiber;
3. The moment capacity of a hybrid NC-UHPC beam used in the experimental program (150 × 150 mm) with a 20 mm to 50 mm thick UHPC layer was higher by 55% compared to the plain concrete beam of similar dimensions and span. The average moment capacity of the hybrid section (9 kN·m) was also close to the moment capacity of the NC beam reinforced with steel bars (10.7 kN·m);
4. The hybrid NC-UHPC beam behaves linearly elastic up to cracking. This measured strain shows a linear behavior confirming that no bond slip occurs at the UHPC and NC interface in the hybrid beam. The linear behavior allows the usage of the transformed section to evaluate deflection, stress, and flexural bearing capacity. The UHPC layer needs to be partially cured with an unfinished top surface for at least 48 h first, and then the normal concrete is placed over it to increase the bond between both layers and consequently develop a complete composite system without any bond slip at the interface;
5. The measured deflection in the hybrid beam at cracking can be computed with a reasonable accuracy using the moment of inertia for a cracked section in which the UHPC layer is cracked to a great extent. The difference between measured and computed deflections ranges from 0.2% to 31%.

## Figures and Tables

**Table 1:** Physical properties of OPC, MS, FA, and CA.

Properties	OPC	MS	FA	CA
**Physical**				
Bulk density (kg/m^3^)	3150	395	1540	1600
Absorption (%)	-	-	0.4	1.0
Specific gravity	3.15	2.25	2.53	2.67
Specific area (cm^2^/gm)	3200	20,000	-	-
Fineness modulus	-	-	2.22	7.11
Average particle size (μm)	1.64	0.142	600	10–20 mm
Color	Grey	Light grey	-	-
**Chemical compositions (%)**				
SiO_2_	22	92.5	-	-
Al_2_O_3_	5.64	0.72	-	-
Fe_2_O_3_	3.8	0.96	-	-
CaO	64.35	0.48	-	-
MgO	2.11	1.78	-	-
SO_3_	2.1	0.15	-	-
K_2_O	0.36	0.84	-	-
Na_2_O	0.19	0.5	-	-
LOI	0.7	1.55	-	-

**Table 2:** Steel fiber details.

Details	Copper-Coated Steel Fiber
Type	Straight
Length	13 mm
Diameter	0.2 mm
Aspect ratio (L/D)	65
Tensile strength	2500 MPa

**Table 3:** Mixture Proportions of UHPC and NC (kg/m^3^).

Type	OPC	MS	FA	CA	Steel Fibers	Superplasticizer	Water
UHPC	900	220	990	-	157	42	168
NC	400	-	729	1092	-	-	184

**Table 4:** Details of hybrid NC-UHPC beams.

Designation	Hybrid Beam Dimensions (mm), See Figure 3			UHPC Thickness, (t) (mm)	Testing Dimensions (mm), See Figure 3		a/h Ratio
	b	h	L		Testing Span	Shear Span, (a)	
A20	150	150	760	20	630	240	1.6
A40				40			
B20	150	150	1000	20	900	375	2.5
B40				40			
C25	150	200	900	25	750	300	1.5
C50				50			
D25	150	200	1200	25	1100	475	2.4
D50				50			

**Table 5:** Test results of UHPC and NC used in hybrid beams.

	Test	Specimens Size	Results (Average of 3 Specimens) (MPa)
**UHPC**	Compressive strength	50 mm cube	172
	Compressive strength	75 mm dia. × 150 mm length (cylinder)	160
	Elastic Modulus	75 mm dia. × 150 mm length (cylinder)	55,000
	Direct tensile strength	Dog-Bone Test Specimen (ASTM D638)	10
	Flexural strength	40 mm × 40 mm × 160 mm (prism)	27
	Flexural strength (No fibers)	40 mm × 40 mm × 160 mm (prism)	13
	Splitting tensile strength	75 mm dia. × 150 mm length (cylinder)	15
**NC**	Compressive strength	100 mm cube	45
	Compressive strength	75 mm dia. × 150 mm length (cylinder)	40
	Elastic Modulus	75 mm dia. × 150 mm length (cylinder)	30,000

**Table 6:** Hybrid beams spans, failure load, and moment capacity.

Specimens	Testing Span (mm)	Shear Span (a) (mm)	Shear Span/Total Depth (a/h)	Shear Span/UHPC Thickness (a/t)	Average Experimental Cracking Load (kN)	Average Failure Load (kN)	Average Moment Capacity kN·m
A20	630	240	1.6	12	45	52	6.24
A40				6	51	70	8.4
B20	900	375	2.5	18.8	20	20	3.8
B40				9.4	31	34	6.4
C25	750	300	1.5	12	64	70	10.5
C50				6	70	90	13.5
D25	1100	475	2.4	19	28	28	6.7
D50				9.5	38	46	10.9

**Table 7:** Experimental results of load and strain for beams A20 and B20.

Hybrid Beam ID	P_cr_. (kN)	Level of Load (kN)	% of P_U_	Measured Strain × 10^−6^				Measured NA from Top mm
				Top Side	1/3rd from Top	2/3rd from Top	Bottom Side	
A20	45	12.3	23	−58	−24	8	54	81
		24.2	45	−119	−47	20	111	81
		36.6	70	−119	−70	36	262	72
		42.5	80	−231	−80	56	300	69
		48.4	90	−283	−83	112	350	65
B20	20	4.9	25	−44	−19	11	39	81
		10.3	50	−99	−38	30	91	78.1
		15.2	75	−155	−55	65	166	72.6
		19.6	98	−225	−72	110	255	68

**Table 8:** Experimental results of load and strain for beams D25 and D50.

Hybrid Beam ID	P_cr_. (kN)	Level of Load (kN)	Percentage of P_U_	Measured Strain × 10^−6^				Measured NA from Top, mm
				Top Side	1/3rd from Top	2/3rd from Top	Bottom Side	
D25	28	7.2	25	−38	−18	13	42	105
		14.5	50	−106	−38	31	92	105
		21.5	75	−158	−57	63	110	98
		28.3	100	−249	−57	110	233	86
D50	38	11.3	25	−75	−30	19	64	108
		22.6	50	−120	−63	40	132	107
		33.9	75	−217	−100	71	173	109
		39.3	85	−282	−120	55	205	111

**Table 9:** Calculated NA depth and MI of hybrid beams.

Hybrid Beam ID	Uncracked		Cracked NC Layer		Cracked UHPC	
	NA from Top (mm)	I_uc_ × 10^6^	NA from Top (mm)	I_crn_ × 10^6^	NA from Top (mm)	I_cru_ × 10^6^
		(mm^4^)		(mm^4^)		(mm^4^)
A20	81.5	51.8	71.1	44.3	68.0	31.9
A40	85.0	55.2	83.0	54.4	61.8	25.3
B20	81.5	51.8	71.1	44.3	68.0	31.9
B40	85.0	55.2	83.0	54.4	61.8	25.3
C25	108.3	121.8	93.1	102.0	91.3	77.0
C50	112.9	130.4	109.5	127.5	83.4	61.5
D25	108.3	121.8	93.1	102.0	91.3	77.0
D50	112.9	130.4	109.5	127.5	83.4	61.5

**Table 10:** Moment capacity of hybrid, plain NC, and reinforced NC sections.

Hybrid Beam ID	Exp. Cracking Load (kN)	Exp. Moment Capacity (kN·m)	Calculated Cracking Load	Calculated Moment Capacity (kN·m)	Moment Capacity of Plain NC Section (kN·m)	Moment Capacity of Reinforced NC Section (kN·m)
			(kN)			
A20	45	5.4	38	4.6	3.4	7.5
A40	51	6.1	42	5.0	3.4	7.5
B20	20	3.8	24	4.5	3.4	7.5
B40	31	5.8	27	5.1	3.4	7.5
C25	64	9.6	53	8.0	6.0	10.7
C50	70	10.5	60	9.0	6.0	10.7
D25	28	6.7	34	8.1	6.0	10.7
D50	38	9.0	38	9.0	6.0	10.7

**Table 11:** Measured and Calculated deflection of hybrid beams.

Hybrid Beam ID	Measured Deflection (mm)	Calculated Deflection I_un_ (mm)/%Error		Calculated Deflection I_crn_ (mm)/%Error		Calculated Deflection I_cru_ (mm)/%Error	
A20	0.19	0.117	−61.75	0.137	−38.28	0.190	0.21
A40	0.27	0.122	−121.83	0.124	−118.32	0.266	−1.60
B20	0.38	0.225	−68.57	0.264	−44.11	0.365	−3.99
B40	0.4	0.238	−68.24	0.242	−65.58	0.519	22.94
C25	0.25	0.120	−107.79	0.144	−73.92	0.190	−31.28
C50	0.31	0.125	−147.73	0.128	−142.23	0.265	−16.86
D25	0.4	0.244	−64.14	0.291	−37.38	0.386	−3.70
D50	0.52	0.255	−103.69	0.261	−99.17	0.541	3.92

## Data Availability

The datasets generated during and/or analysed during the current study are available from the corresponding author on reasonable request.
